# A dataset for Moroccan sign language recognition and translation

**DOI:** 10.1016/j.dib.2025.112395

**Published:** 2025-12-16

**Authors:** Ben Zaid Fatima, Benaddy Mohamed, Boukdir Abdelbasset, El Meslouhi Othmane

**Affiliations:** a Research Laboratory LABSI, FSA, Ibno Zohr University Agadir, Morocco; b FPO, Ouarzazate, Ibno Zohr University Agadir, Morocco; c ENSA, Safi, Cadi Ayad University Marrakech, Morocco

**Keywords:** Moroccan sign language (MoSL), Sign language dataset, Sign language recognition, Sign language translation, Deaf communication

## Abstract

This paper introduces a new dataset of Moroccan Sign Language (MoSL) that aims for use in sign language recognition and translation research. The dataset has been created by recording 2199 word-level isolated signs by nine native signers in MP4 video format. It covers a broad spectrum of lexical categories like letters, numbers, pronouns, as well as frequently used day-to- day words. Sign language being a visual-gestural means of communication holds not just linguistic importance but also a cultural as well as regional identity. A language like MoSL that holds such significance constitutes a low-resource language with digital representation that can be scraped barely on currently available linguistic datasets. A solution towards this shortage in publicly available resources was found by offering systematically annotated as well as video file-organized datasets by means of an online repository. MoSL dataset thus counts as a useful dataset that can further computer vision as well as natural language processing task-oriented apps in sign language research leading towards more inclusive communication tools for deaf as well as hard-of-hearing populations.

Specifications Table

**Table:** 

Subject	Computer Sciences
Specific subject area	Moroccan Sign Language (MoSL).
Type of data	Video (.mp4), Text (.csv).
Data collection	This dataset for this research is the Moroccan Sign Language dataset containing 2,199 videos. In order that it was rich in content, of good quality and representative, it was constructed under the contribution of nine sign experts. The records were performed by applying a spatial resolution of 460×460 pixels with a frame rate equal to 25 frames per second (fps).
Data source location	Institution: The Shorouk Association for the Deaf and Hard of Hearing. City/Province: Ouarzazate. Country: Morocco.
Data accessibility	• Repository name: Mendeley Data • Data identification number: 10.17632/23phgyt3mt.1 • Direct URL to data: https://data.mendeley.com/datasets/23phgyt3mt/1
Related research article	None

## 1 Value of the Data

• This dataset was prepared with the Shorouk Association for the Deaf and Hard of Hearing in Ouarzazate. Nine native signers (7 women, 2 men) contributed to the dataset to ensure variety and real representation of Moroccan Sign Language (MoSL).
• The dataset includes basic gestures (letters, numbers, pronouns), temporal vocabulary (days, months, seasons) and everyday nouns and verbs. It’s a complete tool for studying and learning MoSL.
• This dataset provides video recordings and corresponding CSV files of 2,199 gestures of Moroccan Sign Language (MoSL), to aid further the reproducibility of the analyses and experiments. Particularly for researchers, faculty and learners studying MoSL, its format is designed so that they create a systematic format to be used in academic literature review and actual learning projects.
• The dataset can be employed for the research and experimental implementation of automatic sign recognition systems for sign language translation systems, and even for gesture processing.
• The vocabulary comprises signs that are normally taught during first lessons and that are helpful for teaching.
• The dataset is directly connected to image-based and video recognition systems since the data is obtained from video recorded applications. Its practical application is in real-time translation, evaluation systems, interactive learning, mobile devices, humanoid robots, VR/AR applications, as well as educational games.
• Being a public dataset, it can not just be expanded or improved on but also can act as an educational dictionary or reference resource for schools as well as Deaf associations.
• Using it, researchers can create machine learning frameworks for bidirectional sign translation systems such as baseline CNN models or integration with Arabic NLP pipelines for text-to-sign translation, in addition to mobile or web applications for assistive communication and deaf education.

## 2 Background

Sign language is a visual-gestural communication system that utilizes hand movements, facial expressions, and body posture to convey meaning [1,2], It plays a vital role not only for individuals who are deaf or hard of hearing but also for hearing individuals who wish to communicate inclusively. According to the World Health Organization (WHO), over 5 % of the global population around 430 million people, including 34 million children—require rehabilitation for disabling hearing loss [3].

In Morocco, approximately 300,000 individuals live with varying degrees of deafness [4], highlighting the urgent need for accessible communication solutions and the development of structured sign language resources.

In Morocco, deaf children have historically received very limited educational support. For many years, they learned local sign language varieties influenced by Arabic, French, and American Sign Languages [5].

In April 2019, the government officially standardized Moroccan Sign Language (MoSL) and launched programs aimed at improving deaf children’s education [6]. However, most teachers involved are hearing individuals with limited proficiency in MoSL and lack adequate resources and tools to help deaf students learn from written or spoken language. To bridge this gap, schools often hire interpreters to facilitate students’ understanding of classroom instruction. Otherwise, teachers rely on graphics and captioned videos to help students associate concepts with signs, but there remains a lack of tools that directly translate written or spoken words into MoSL.

## 3 Data Description

**Fig. 1:**
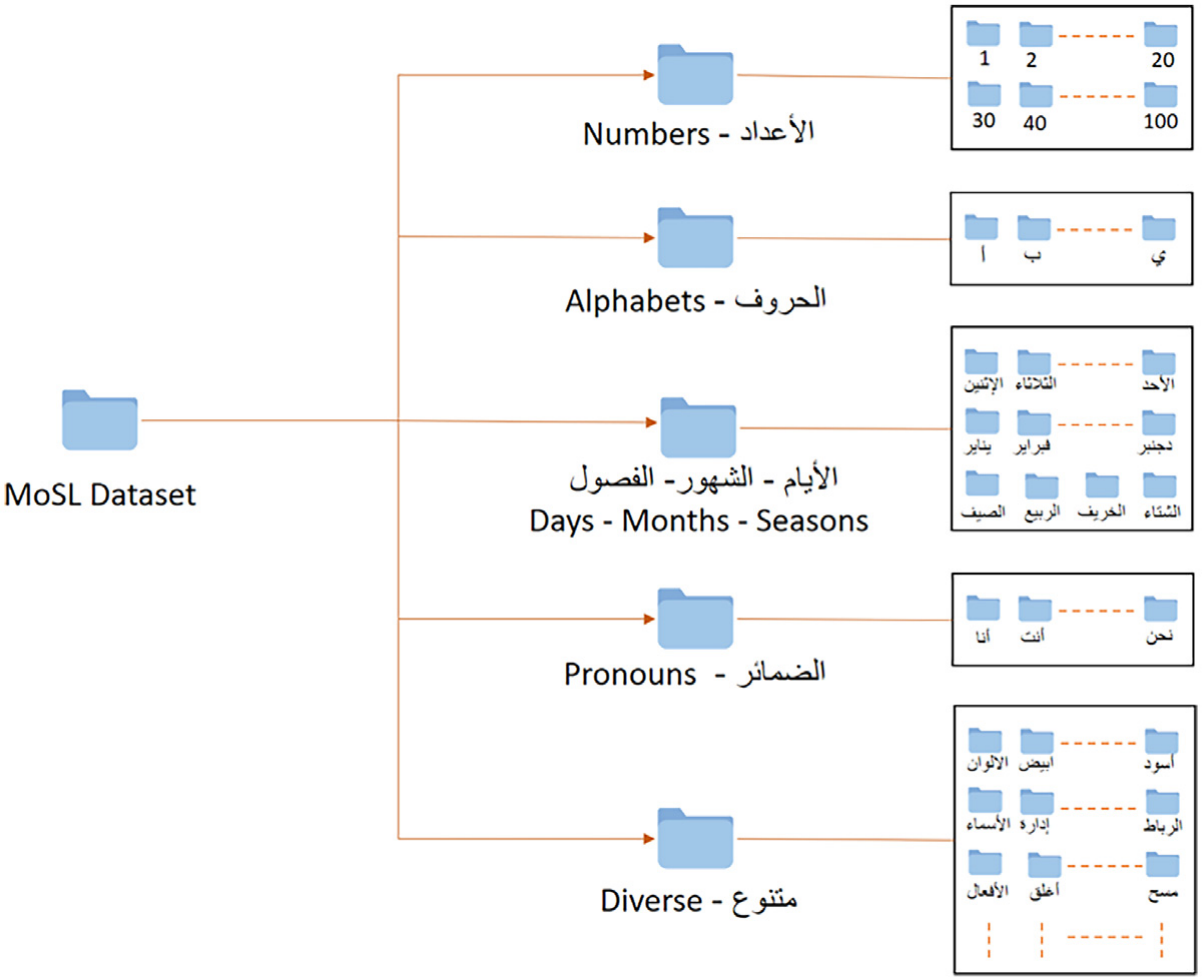
MoSL dataset structure.

• The MoSL dataset consists of standard signs like alphabetic symbols, numbers, pronouns, and Days - Month - Seasons as well as diverse data for everyday life. This dataset is a set of the data used by a Shorouk Association for the Deaf and Hard of Hearing, which is a specialized institution dedicated to the education of students with special needs, particularly those with hearing impairments. The purpose of the dataset is to:
  ○ Create a dataset of basic MoSL sign gestures (e.g., alphabet, numbers, pronouns, etc.) by including the content of simple static and dynamic signs that are publicly available.
  ○ Encourage researchers to work on Moroccan sign language translation.
• Nine volunteers participated in the recording, called signers (2 male; 7 female). The gender of each signer is detailed in Table 1. The dataset contains five folders: alphabet, number, pronouns, Days - Month - Seasons, and Diverse. The directory is shown in Fig. 1; Table 2 displays the directory structure and subfolders further. All 9 signers recorded each sign of alphabet, number, pronouns, and the Days - Month - Seasons sign. The dataset categorizes number signs into static: numbers 1 through 9, and dynamic: a combination of several number sign gestures. For example, the number 11 to 19 sign is a unit gesture performed twice. The fifteen sign combines the signs of one and five. Each video file is saved with a name based on that information. The pronouns directory consists of 15 distinct types in Moroccan, which include singular, dual, and plural forms.

**Table 1:** Demographic summary of MoSL signers.

Attribute	Count	Details Total
Participants	9	2 Males, 7 Female
Age Range	23–38 years	Mean: 30.5 years

**Table 2:** Detailed directory structure.
• The Days-Months-Seasons directory comprises all the days of the week, the months of the year, and the four seasons.
• The divers directory contains a lot of sub-directories representing the basic words using in everyday life: colors, Nouns …
• The sample images in Table 3 illustrate different sign classes captured under varying conditions, including changes in position, lighting, signer identity, and sign type.

**Table 3:** Sample sign images of the dataset.
• This dataset contains five CSV files for all categories specific to Moroccan Sign Language (alphabet, number, pronouns, and Days - Month - Seasons). Each CSV file stores one row per video corresponding to the sign, with extensive metadata about video recording and video presentation. The metadata fields for each video are: video_name, sign_label (in Arabic and English), signer_id, duration_seconds, frame_rate, frame_count, resolution, file_path, file_size_bytes. The same format structures the placement of video files and also the descriptive structure, making it very easy to filter the experiment, preprocess the results, and maintain reproducible results for the data.

## 4 Experimental Design, Materials and Methods

Sign language, like spoken languages, follows its own grammatical rules and structure [7] However, these rules vary significantly across countries and regions. Countries that share a spoken language may still have completely distinct sign languages. For instance, British Sign Language (BSL) and American Sign Language (ASL) are mutually unintelligible, despite both being used in English-speaking countries [8].

Most sign languages are more closely tied to national identity than to the spoken language of a region. This is especially evident in the Arab world. Although Arabic is the common spoken language, each country often uses a different sign language. Arabic Sign Language (ArSL) is thus better understood as a group of related but distinct sign languages rather than a single unified language [9]. For example, Moroccan Sign Language (MoSL) differs from Algerian and Egyptian Sign Languages in both vocabulary and structure .

These linguistic differences highlight the need for country specific datasets and sign language resources. Developing tools tailored to the linguistic and cultural context of a specific country such as MoSL ensures accuracy, accessibility, and relevance for the Deaf community .

Sign language consists of two major types: static gestures and dynamic gestures (also termed ideograms). Dynamic gestures involve coordinated hand movements, interaction between the hands and other parts of the body and include the use of facial expressions to represent semantic content.

The dataset was prepared carefully to get sufficient of these key components including the visual and environmental setup. Standardized recording environment was employed to reduce noise levels and to maximize contrast in visual data. To minimize background noise, a blue background was established and participants were instructed to wear black long-sleeved shirts to hide their arms. Furthermore, this position improves the visibility of hands and face fields that are important for sign recognition. Each sign often starts with the hands lying in a resting position with the body. The gesture is a series of regular motions and ends with the hands at rest once more.

The dataset is equipped with whole time frames corresponding to each performed gesture, for modeling movement and temporal dynamics.

The dataset for generating the significant elements, including gesture translation, employed in Moroccan Sign Language gestures was purposely developed so that its contents was both visual and environmentally appropriate. All recordings were carried out using frame rates of 25 frames per second (fps) and a resolution of 460×460 pixels. The same recording environment was used to minimize noise and maximize contrast in the recording space (in a blue background to minimize interference), and participants were instructed to wear black long-sleeved shirts to bring the hands into sharp focus.

Every sign was executed 1–3 times by native Moroccan Sign Language (MoSL) signers resulting in the variation in execution. Each sign starts with the hands sitting with the body, then runs to an unbroken series of gestures in the hands, and finally concludes with the hands returning to the resting position. Every gesture is depicted in full frame-based order throughout time, and the relevant frames are saved, so they are very accurate for motion and time complexity modeling.

All of the videos were taken in MP4 format and renamed by the following conventions *sign_label.mp4*. Python scripts extracted metadata related to video, such as duration, frame rate, frame count, resolution, and file size, then served these information to structured CSV files. It is broken down into categories and folders (Alphabet, Numbers, Pronouns, Days–Months–Seasons or Diverse). All videos were screened manually, with samples containing hand occlusion or excess motion blur and wrong execution or incomplete gestures filtered out. This uniform picture quality ensured that the folder hierarchy, annotation rules, metadata format, and scripts for automatic extraction are preserved. It is possible to integrate directly with the Sign Language Recognition DL pipeline.

## Limitations

This study acknowledges multiple limitations in both the dataset and the process of data collection. The dataset consists of 2,199 isolated sign words. Although the videos lack audio, there are visible lip movements for future research in lip-reading.

The dataset was obtained from a limited number of participants which demonstrated the imbalance in the representation of the sexes between male and female signers.

This dataset consists of basic root words, pronouns, numbers and alphabet signs, future expansion will involve more complex sentence structures.

Nonetheless, this dataset contributes greatly to the study of Moroccan Sign Language (MoSL), though its shortcomings are well documented.

In future efforts, it will be our goal to enhance the collaboration with a diversity of MoSL experts. Not only to broaden the data pool, but also to achieve more participation of the community in the research process. Also, we intend to incorporate advanced translation technologies from Neural Machine Translation (NMT), to improve the quality of translation between Moroccan spoken language and MoSL. We will also utilize gesture and movement features to optimize the translation process and achieve greater accuracies in converting spoken Moroccan language to MoSL.

## Ethics Statement

Although the videos were provided by the Shorouk Association for the Deaf and Hard of Hearing, we have provided links to the videos in the dataset only, without distributing them to the public repository.

## CRediT Author Statement

**Ben zaid fatima**: Conceptualization, Methodology, Data Curation, Investigation, Writing- Original Draft, Review & Editing; **Benaddy mohamed**: Conceptualization, Methodology, Data Curation, Investigation, Writing - Original Draft, Review & Editing; **Boukdir abdelbasset**: Conceptualization, Methodology, Data Curation, Investigation, Writing - Original Draft, Review & Editing; **EL meslouhi othmane**: Writing – review & editing, Investigation.

## Data Availability

Mendeley DataA Dataset for Moroccan Sign Language (MoSL) (Original data).
